# Supplementary material for: Chromothripsis during telomere crisis is independent of NHEJ, and consistent with a replicative origin
Source: Genome Res. 2019 May;29(5):737–49. doi: 10.1101/gr.240705.118 (PMC6499312; doi:10.1101/gr.240705.118)
Supplement: Supplemental Material [file supp_gr.240705.118_Supplemental_file_1.zip › contigs/annotated_contigs/DB47/contig.2.DB47_length_407_mean_cov_3.31695331695.docx]

**DB47_length_407_mean_cov_3.31695331695**

```
GCTACCCATATCCACCAAGACAAGCCCCCATCAGTTATATTTAGTGTACTGTCTCAGTACATTTACACAGTACATCTGTGAAAACACAA
 >chr4:148451681-148451941 - E=4e-145
CTCTTAGAATTATGTTGACTGTTTCTTCACCAGAATTCCCTAGAGATGAGCATGTACAGATTTGGGTATTTTACGATGATGCCACATGG

CATATTATATAGCACAATTTACTGGAGAAATGTTTTATAAAAGATTTTTAGGGTAAATAAGATTTGTGTACAATACAAATCT|TTTG|T

CCAACCTGGACAAAAGGGCGAAACTCCGTCTCAAAAAAAAAAAAAAA|AACAAGGAGGTAAGGGCGGTGGGGGGGGTGGTGCGGGGCGG
>chr4:148696059-148696107 - E=2e-17
TGAAAGAAAAAAAAGTTTTTTTATTTCCTCCGAGGATCCTTTTCCCCAGAGGTC
```
